# Maternal–Infant Factors in Relation to Extracellular Vesicle and Particle miRNA in Prenatal Plasma and in Postpartum Human Milk

**DOI:** 10.3390/ijms25031538

**Published:** 2024-01-26

**Authors:** Meghan E. Muse, David A. Armstrong, Anne G. Hoen, Diane Gilbert-Diamond, Jiang Gui, Thomas J. Palys, Frederick W. Kolling, Brock C. Christensen, Margaret R. Karagas, Caitlin G. Howe

**Affiliations:** 1 Department of Epidemiology, Geisel School of Medicine at Dartmouth, 1 Medical Center Dr, Lebanon, NH 03755, USAmargaret.r.karagas@dartmouth.edu (M.R.K.); caitlin.g.howe@dartmouth.edu (C.G.H.); 2 Research Service, V.A. Medical Center, Hartford, VT 05009, USA; 3 Department of Dermatology, Dartmouth Health, Lebanon, NH 03756, USA; 4 Department of Biomedical Data Science, Geisel School of Medicine at Dartmouth, Lebanon, NH 03756, USA; 5 Dartmouth Cancer Center, Geisel School of Medicine at Dartmouth, Lebanon, NH 03756, USA; 6 Department of Molecular and Systems Biology, Geisel School of Medicine at Dartmouth, Lebanon, NH 03756, USA

**Keywords:** extracellular vesicles and particles, miRNA, pregnancy, plasma, human milk

## Abstract

MicroRNAs (miRNA) in extracellular vesicles and particles (EVPs) in maternal circulation during pregnancy and in human milk postpartum are hypothesized to facilitate maternal–offspring communication via epigenetic regulation. However, factors influencing maternal EVP miRNA profiles during these two critical developmental windows remain largely unknown. In a pilot study of 54 mother–child dyads in the New Hampshire Birth Cohort Study, we profiled 798 EVP miRNAs, using the NanoString nCounter platform, in paired maternal second-trimester plasma and mature (6-week) milk samples. In adjusted models, total EVP miRNA counts were lower for plasma samples collected in the afternoon compared with the morning (*p* = 0.024). Infant age at sample collection was inversely associated with total miRNA counts in human milk EVPs (*p* = 0.040). Milk EVP miRNA counts were also lower among participants who were multiparous after delivery (*p* = 0.047), had a pre-pregnancy BMI > 25 kg/m^2^ (*p* = 0.037), or delivered their baby via cesarean section (*p* = 0.021). In post hoc analyses, we also identified 22 specific EVP miRNA that were lower among participants who delivered their baby via cesarean section (*Q* < 0.05). Target genes of delivery mode-associated miRNAs were over-represented in pathways related to satiety signaling in infants (e.g., CCKR signaling) and mammary gland development and lactation (e.g., FGF signaling, EGF receptor signaling). In conclusion, we identified several key factors that may influence maternal EVP miRNA composition during two critical developmental windows, which should be considered in future studies investigating EVP miRNA roles in maternal and child health.

## 1. Introduction

MicroRNAs (miRNA) are key epigenetic regulators that post-transcriptionally regulate gene expression. In addition to influencing mRNA expression within cells, miRNA play critical roles in mediating intercellular communication, as they can be transported via extracellular vesicles and other extracellular particles (EVPs) to distal target tissues [1]. In addition to facilitating intercellular communication, EVPs and their miRNA cargo facilitate maternal–offspring communication. During pregnancy, this occurs via the placenta, the interface of maternal-fetal communication [2]. During the postnatal period, this can also occur via transfer in human milk [3,4].

Circulating EVPs originate from multiple cells and tissues. During pregnancy, a large fraction of EVPs in maternal circulation are of placental origin, and the levels of these placenta-derived EVPs increase in maternal circulation over the course of pregnancy [5,6]. MiRNA profiles within maternal circulating EVPs also change during pregnancy [7] and have been associated with both maternal exposures during pregnancy and birth outcomes, such as low birth weight and shorter gestation, which, in turn, influence child growth [8,9,10,11,12,13]. Given that the in utero period is critical for shaping the future health of the developing fetus [14], epigenetic dysregulation during this period, including alterations in maternal–fetal communication via EVP miRNA, has the potential for long-lasting health impacts for the child. Additionally, given the minimally invasive nature of maternal blood collection during pregnancy, circulating EVP miRNAs have the potential to serve as early biomarkers of pregnancy complications and offspring health.

MiRNAs are abundant in human milk and thought to primarily originate in the mammary epithelium [3,4]. In vitro evidence supports the idea that milk-derived EVP miRNA survive conditions mimicking infant digestion [15], and studies using animal models have reported that these miRNAs may also reach offspring circulation and peripheral tissues [16,17]. Therefore, the postnatal period is another sensitive window during which maternal-derived miRNAs may regulate gene expression and developmental programming in the offspring. In addition to their epigenetic roles, it has been hypothesized that milk-derived EVP miRNAs may provide nutritional value to the offspring [18,19,20]. Although studies investigating the potential roles of these miRNAs in offspring health and development are currently limited, recent evidence suggests that they may influence offspring growth and health [21,22].

Currently, little is known about the factors influencing EVP miRNA in maternal plasma during pregnancy or in human milk postpartum. However, identifying such factors is essential for effectively addressing potential confounding and ensuring that future studies of maternal EVP miRNAs are reproducible and biologically meaningful. In addition to clinical factors, a better understanding of the technical factors related to sample collection and processing that may influence EVP miRNA levels is important. For example, recent evidence suggests that EVP release is influenced by circadian rhythms, indicating that time of day of sample collection may be important [23,24,25]. However, few studies have examined how the time of day of sample collection influences miRNA within EVPs, and this has been especially understudied in human populations during pregnancy and postpartum.

In a pilot study of 54 mother–infant pairs in the New Hampshire Birth Cohort Study, we profiled EVP miRNA in paired second trimester maternal plasma and 6-week postpartum human milk samples, comparing the composition between sample types and assessing their relationships with maternal and pregnancy characteristics, as well as the timing of biospecimen collection. We examined whether maternal age, pre-pregnancy BMI, parity, gestational age at sample collection, time of day of sample collection, and infant sex influence maternal plasma EVP miRNA levels during pregnancy. We additionally evaluated whether maternal age, pre-pregnancy BMI, gestational weight gain, parity, infant sex, gestational age at delivery, delivery mode, infant age at sample collection, and time of day of sample collection influence EVP miRNA levels in human milk during the postpartum period.

## 2. Results

### 2.1. Participant Demographics

The study’s participants were, on average, in their early thirties (mean age 32.56 ± 4.0 years), and approximately 87% of participants were college graduates (Table 1). There were slightly more female (55.6%) than male infants, and 74.1% of infants in this study were delivered vaginally.

### 2.2. EVP miRNA Composition of Maternal Plasma during Pregnancy and Paired Human Milk Postpartum

In total, 11 miRNAs were above the background threshold in 100% of maternal plasma samples, and 76 miRNAs were above the background threshold in 100% of milk samples; nine of these miRNAs were detected in 100% of both sample types (Table S1). Of the 142 miRNAs above the background threshold for >60% of maternal plasma samples and 200 miRNAs above the background threshold for >60% of milk samples, 92 were detectable in >60% of both sample types.

**Figure 1:**
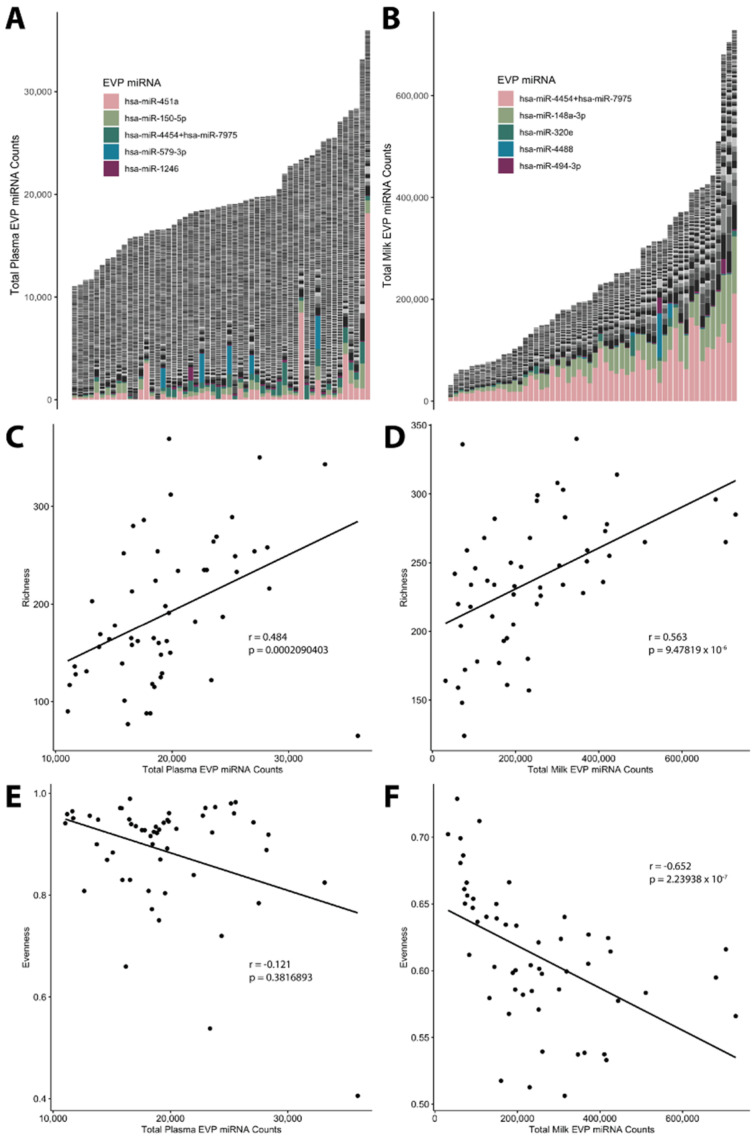
EVP miRNA composition for (**A**) plasma samples (n = 54) and (**B**) milk samples (n = 54). Scatterplots showing relationships between total counts and sample richness (i.e., the number of unique miRNA transcripts detected in each sample) for (**C**) plasma and (**D**) milk samples with corresponding correlations. Scatterplots showing relationships between total counts and sample evenness for (**E**) plasma and (**F**) milk samples with corresponding correlations. Sample evenness is bound between 0 and 1, where 1 reflects equal distribution in the relative abundance of all detectable miRNA transcripts in a given sample. Correlations were calculated using the Spearman correlation coefficients.

MiRNAs represented by three Nanostring probes (hsa-miR-451a, hsa-miR-4454 + hsa-miR-7975, and hsa-miR-579-3p) comprised ≥10% of total miRNA counts for at least one plasma sample (Figure 1A), while miRNAs represented by five Nanostring probes (hsa-miR-4454 + hsa-miR-7975, hsa-miR-148a-3p, hsa-miR-320e, hsa-miR-4488, and hsa-miR-494-3p) comprised ≥10% of total counts for at least one milk sample (Figure 1B). Sample richness, i.e., the number of unique miRNA transcripts detected in each sample, was positively correlated with total miRNA counts in both plasma (*r* = 0.484, *p* < 0.001; Figure 1C) and milk (*r* = 0.563, *p* < 0.001; Figure 1D). Of the 798 miRNAs measured, 156 miRNAs were positively associated with sample richness in plasma (Figure S1; Table S2), and 105 were positively associated with sample richness in human milk (Figure S2; Table S3).

Although total miRNA counts were not significantly correlated with sample evenness, a measure of the uniformness in the distribution of the relative abundance of all detectable EVP miRNAs in a sample, in plasma (*r* = −0.121; *p* = 0.381; Figure 1E), they were negatively correlated with sample evenness in milk (*r* = 0.652, *p* < 0.001; Figure 1F). Of the 798 miRNAs measured, 55 were associated with sample evenness in plasma (Figure S1; Table S1), and 77 were associated with sample evenness in milk (Figure S2; Table S2).

### 2.3. Time of Day of Blood Collection Is Associated with Maternal Plasma EVP miRNA Composition during Pregnancy

**Figure 2:**
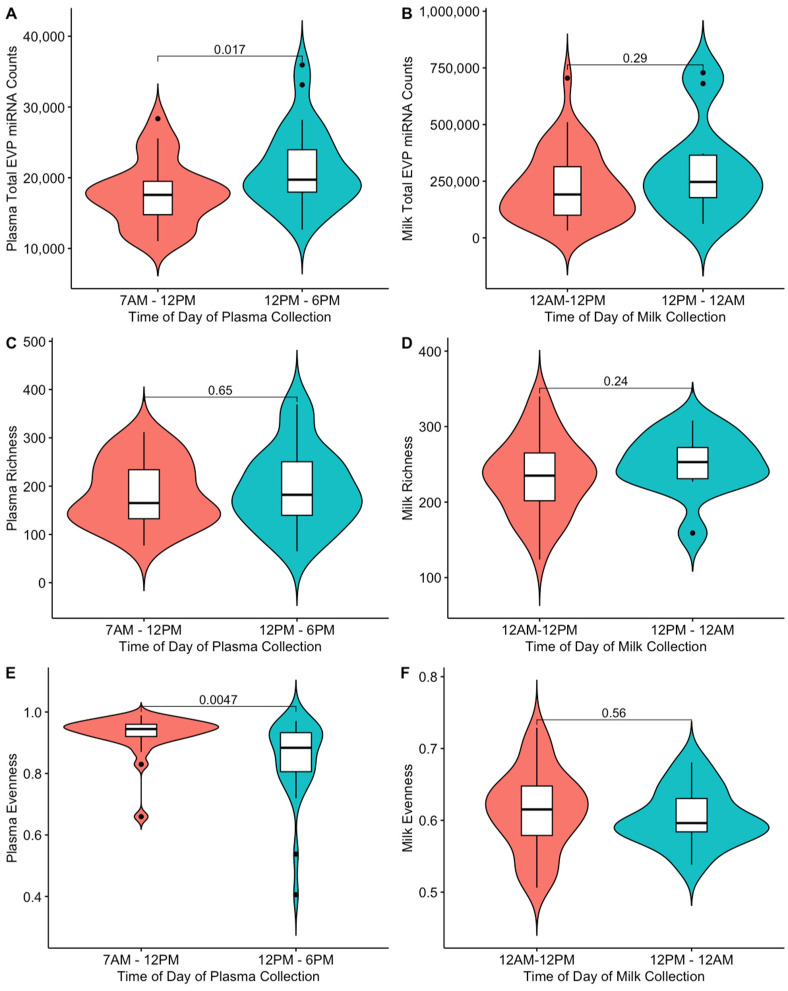
MiRNA composition by timing of sample collection: (**A**) total counts in plasma, (**B**) total counts in milk, (**C**) plasma sample richness, (**D**) milk sample richness, (**E**) plasma sample evenness, and (**F**) milk sample evenness. *p* values were obtained from a Wilcoxon Rank-Sum test. Collection time is missing for two milk samples in which subjects did not provide a time with their sample.

In bivariate analyses for plasma, we observed associations with a *p* < 0.10 between the time of day of blood collection and both total miRNA counts and sample evenness (Figure 2), between maternal age and sample richness, and between parity and total miRNA counts (Figure S3; Table S4).

Total miRNA counts were higher for blood samples collected in the afternoon compared with the morning (0.234 log_2_-fold difference; 95% CI: 0.037, 0.430; *p* = 0.024), adjusting for maternal age and parity (Table 2). Blood samples collected in the afternoon also had a lower (−0.093; 95% CI: −0.148, −0.038; *p* = 0.002) sample evenness score compared with samples collected in the morning. No associations were observed between the time of day that the sample was collected and sample richness. All findings were consistent in sensitivity analyses, restricting analysis to participants who reported a history of never smoking (n = 51) and excluding participants with gestational diabetes (n = 3) and participants with pre-eclampsia (n = 4; Table S6).

In post hoc analyses focusing on evenness-associated miRNA, the odds of detecting both hsa-miR-423-5p (OR = 0.180; 95% CI: 0.048, 0.679; *p* = 0.011) and hsa-miR-587 (OR = 0.244; 95% CI: 0.0734, 0.810; *p* = 0.021) in plasma EVPs were higher for samples collected in the afternoon compared with the morning (*p* < 0.05), but this was not statistically significant after multiple testing correction (Table S7).

### 2.4. Delivery via Cesarean Section Is Associated with Lower Total EVP miRNA Counts in Milk and Higher Sample Evenness

In bivariate analyses for milk, we observed associations with a *p* < 0.10 between infant age at sample collection, parity, and infant sex and total miRNA counts as well as between gestational weight gain and infant sex and sample evenness, (Figure S4; Table S5).

Total miRNA counts in milk EVPs were lower for participants who had delivered their baby via cesarean section (−0.703 log_2_-fold difference; 95% CI: −1.28, −0.125; *p* = 0.021; Table 3) compared with participants who delivered their baby vaginally. Delivery via cesarean section was also associated with a higher sample evenness score (0.040, 95% CI: 0.009, 0.071; *p* = 0.015) compared with vaginal delivery. These findings were consistent in sensitivity analyses restricted to only participants who reported a history of never smoking (n = 51), excluding participants with gestational diabetes (n = 3) and participants with pre-eclampsia (n = 4; Table S8). 

**Figure 3:**
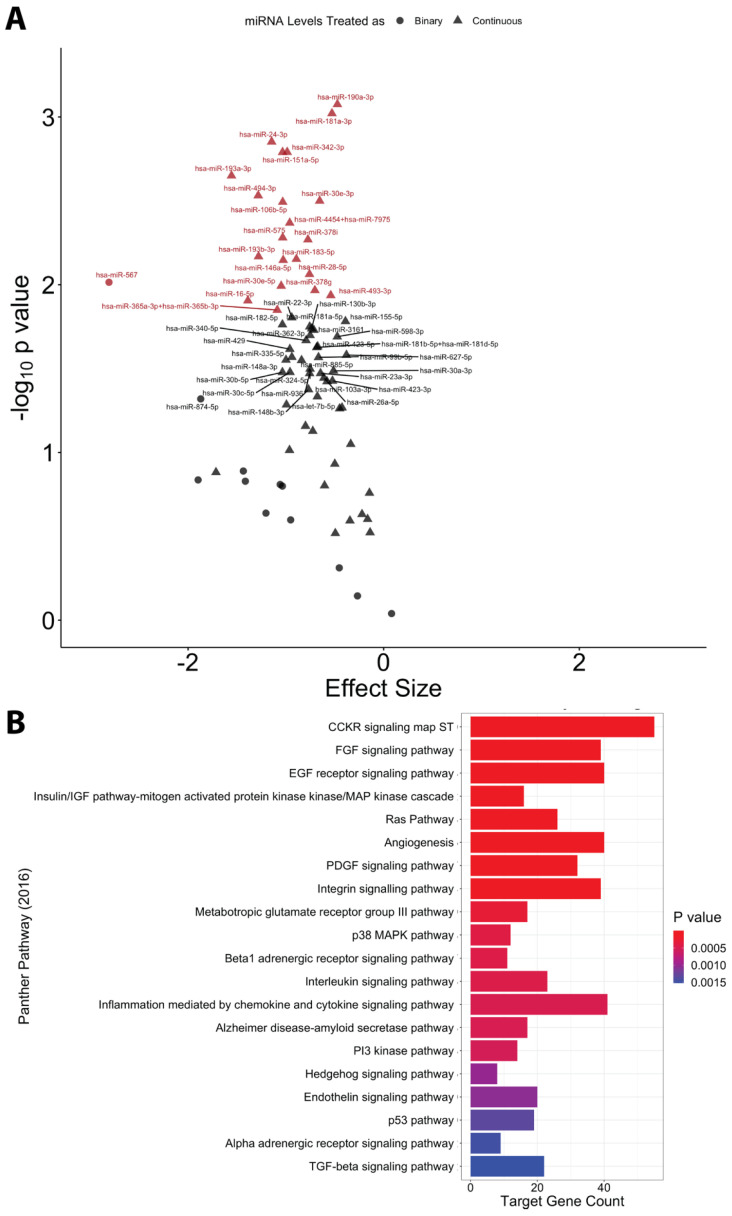
(**A**) Volcano plot showing covariate-adjusted associations between cesarean section delivery (relative to vaginal delivery) and evenness-associated miRNAs in milk EVPs. Labeled points reflect miRNAs that met a nominal significance threshold (*p* < 0.05). Red points reflect miRNAs that reached statistical significance after FDR adjustment (*Q* < 0.05). EVP miRNAs treated as binary variables (above versus below the limit of detection) were modeled using robust logistic regression. EVP miRNA treated as continuous variables were log_2_-transformed and modeled using robust linear regression. All models were adjusted for parity, infant sex, gestational weight gain, and infant age at sample collection. (**B**) A total of 2016 Panther pathways enriched for the 2584 high-confidence target genes identified via the microRNA Data Integration Portal (mirDIP) for the 22 miRNA in milk that were lower among individuals who experienced a cesarean section delivery (*Q* < 0.05).

In a post hoc analysis focusing on the 77 miRNAs that were associated with the milk sample evenness score, 22 were significantly (*Q* < 0.05) lower in the milk of participants who delivered their baby via cesarean section (Figure 3A; Table S9). Observed associations with these 22 miRNAs were consistent in sensitivity analyses restricted to participants who reported a history never smoking (n = 51), excluding participants with gestational diabetes (n = 3) and participants with pre-eclampsia (n = 4; Table S10). Using mirDIP, a total of 2585 unique mRNA transcripts were identified as high-confidence target genes for these 22 miRNAs. A total of 39 PANTHER (2016) pathways were significantly enriched (*P_Benjamini-Hochberg_* < 0.05) for these target genes. Of these 39 pathways, the top three were the CCKR signaling map ST pathway, FGF signaling pathway, and EGF receptor signaling pathway (*P_Benjamini-Hochberg_* < 0.001; Figure 3B).

### 2.5. Parity, Pre-Pregnancy Weight Status, and Infant Age at Sample Collection Are Negatively Associated with Total miRNA Counts in Human Milk EVPs

Total counts in human milk EVPs were lower among multiparous individuals compared with primiparous individuals after delivery (−0.526, 95% CI: −1.032, −0.019; *p* = 0.047) (Table 3). Similarly, total counts were lower among participants with a pre-pregnancy BMI greater than 25 kg/m^2^ compared with participants who had a pre-pregnancy BMI of 18.5–25 kg/m^2^ (−0.645, 95% CI: −1.232, −0.058; *p* = 0.037). Additionally, infant age (days) was inversely associated with total miRNA counts in milk EVPs (−0.049, 95% CI: −0.095,−0.004; *p* = 0.040). These associations were direction-consistent in sensitivity analyses, restricting to participants who reported a history of never smoking (n = 51; Table S8).

We did not identify any statistically significant associations between pre-pregnancy BMI, pre-pregnancy weight status, parity, and infant age at sample collection with sample richness or evenness scores in milk (Table 3).

## 3. Discussion

To the best of our knowledge, this is the first study to characterize EVP miRNAs in paired maternal plasma samples collected during pregnancy and human milk samples collected postpartum. In this study, we examined maternal, pregnancy, and sample collection characteristics as potential predictors of maternal prenatal plasma and postpartum human milk EVP miRNA composition. We found the time of day of blood collection to be a predictor of plasma EVP miRNA composition, while delivery mode, maternal pre-pregnancy weight status, parity, and infant age at sample collection were predictors of human milk EVP miRNA composition.

Given that miRNA in maternal circulation during pregnancy and in human milk postpartum are both thought to play important roles in maternal–offspring communication and offspring development, we compared maternal EVP miRNA profiles across these two sample types. Overall, miRNA composition differed substantially between maternal prenatal plasma and postpartum human milk. Individuals with more abundant levels of EVP miRNAs in their plasma during pregnancy reflected a larger number of unique miRNAs, whereas individuals with more abundant levels of EVP miRNA in human milk reflected a smaller number of distinct miRNAs (e.g., hsa-miR-4454 + hsa-miR-7975, hsa-miR-148a-3p, hsa-miR-320e, hsa-miR-4488, and hsa-miR-494-3p). Prior studies have reported more abundant levels of hsa-miR-320e and hsa-miR-148a-3p in milk EVPs from mothers with HIV-1 compared with uninfected mothers [26]. Studies using mouse models have also demonstrated that EV-derived hsa-miR-148a-3p from human milk may protect against necrotizing enterocolitis [27], suggesting that these miRNA may have important immunoregulatory roles in infants. Interestingly, hsa-miR-4454 and hsa-miR-7975, which are measured using a shared NanoString probe due to sequence similarities in the probed regions of the miRNAs, were highly abundant in both the plasma and human milk samples in our study. Previous studies of pregnant individuals have reported associations between plasma levels of both hsa-miR-4454 and hsa-miR-7975 and gestational age at birth [12]. In the present study, we did not observe evidence that gestational age at birth is associated milk EVP miRNA composition, and associations with plasma EVP miRNA composition were not assessed given that the focus of this paper was predictors of EVP miRNA composition. A prior study also reported lower levels of hsa-miR-7975 in the mature milk of mothers who delivered prematurely compared with mothers who delivered at term, which suggests that circulating levels of this miRNA may influence gestational age at birth and milk levels of the same miRNA may be sensitive to gestational age at birth [28,29]. Hsa-miR-4454 and hsa-miR-7975 have also been associated with infant birth weight [30]. Additionally, during childhood, plasma levels of hsa-miR-4454 have been associated with BMI [31,32], and its downstream target genes are enriched in the insulin receptor signaling pathway and have been hypothesized to regulate the cellular response to insulin through the modulation of alternative splicing [33]. Hsa-miR-4454 may, therefore, play important roles in early growth and metabolic programming [26,27].

Although EVP miRNAs are hypothesized to play critical roles in offspring development both in utero and during the postnatal period, we found that the specific miRNAs present in each sample type were largely distinct. This suggests that different miRNAs may be critical during each of these unique developmental windows. For example, while we identified 11 miRNAs that were present in 100% of the plasma samples during pregnancy and 76 miRNAs that were present in 100% of the milk samples postpartum, only nine of these miRNAs (hsa-miR-1253, hsa-miR-1285-5p, hsa-miR-23a-3p, hsa-miR-302d-3p, hsa-miR-3144-3p, hsa-miR-320e, hsa-miR-378e, hsa-miR-379-5p, and hsa-miR-548ar-5p) were common between the two sample types. This is not surprising, as miRNAs in prenatal plasma and miRNAs in postpartum human milk are thought to have different tissue sources, with the placenta being a major source of circulating miRNA during pregnancy and the mammary epithelium being the main source of miRNA in human milk [3,4,5]. Of these shared, ubiquitously expressed EVP miRNAs, several have important roles in immune function or have been implicated in pregnancy complications (e.g., hsa-miR-302d-3p, hsa-miR-320e, hsa-miR-1253, hsa-miR-378e, has-miR-1253) [34,35,36,37]. These common miRNAs may, therefore, serve important regulatory purposes for child development and growth across these two windows. However, additional work is needed to further assess how the presence of these miRNA in maternal EVPs relates to offspring outcomes, such as growth trajectories and obesity in childhood. This has been particularly understudied for human milk, as prior studies examining relationships between milk EVP miRNAs and infant body composition used candidate gene approaches and, thus, did not evaluate many of the miRNAs that we and others have found to be consistently abundant in human milk [21,22].

In evaluating potential predictors of EVP miRNA composition, we found that the time of day of blood collection was associated with both total miRNA counts and sample evenness in plasma EVPs during pregnancy. This finding is consistent with emerging evidence that there is diurnal variation in both EVP release and EVP cargo and highlights the importance of accounting for the time of day of blood collection in studies of circulating EVP miRNAs during pregnancy [23,24,38]. In contrast with plasma, we did not observe an association between the time of day of milk collection and EVP miRNA composition, consistent with a prior study of bovine milk [39]. Although a previous study of predominantly Hispanic participants in Los Angeles also reported that the levels of many miRNAs change in maternal circulation across gestation [7], we did not observe an association between gestational age at plasma collection and EVP miRNA composition in this pilot study. This could be due to our focus on a much narrower window of pregnancy (~24–28 weeks gestation) compared with this prior study (~14–32 weeks gestation). It is also possible that we were underpowered to detect small changes in miRNA levels during this short gestational window.

Interestingly, we found cesarean section delivery to be associated with the most pronounced differences in milk EVP miRNA composition. After accounting for multiple testing stages, 22 miRNAs were lower among individuals who delivered their baby via cesarean section compared with individuals who experienced a vaginal delivery. It is well known that delivering via cesarean section can lead to delays in the production of mature milk, and the delivery mode has previously been associated with alterations in nutrient levels and the microbiota of human milk [40,41,42,43,44,45,46]. Although very little is known about the impacts of delivery mode on human milk miRNA composition, one small prior study of preterm infants (n = 31) reported statistically significant correlations between a select number of gestational age-associated milk EVP miRNAs and delivery mode in preterm infants but did not investigate this in infants delivered at term [28]. A previous study of colostrum also reported that delivery via cesarean section was associated with two of the same miRNAs (miR-16-5p and miR-30e-5p) that were associated with the delivery mode in our study, although in the opposite direction [47]. To the best of our knowledge, our study is the first to examine the association between the delivery mode and miRNA levels in mature human milk from individuals who delivered at term or post-term. It is, therefore, possible that some of the inconsistencies with these prior studies are due to the different lactation stages evaluated or differences in milk composition for mothers who delivered early versus at or post-term, which merits additional investigation [26]. In pathway analyses, we found that target genes of the delivery-mode-associated miRNAs were over-represented in pathways that are important for infant development, mammary gland development, and lactation. For example, the top pathway identified was CCKR signaling, which regulates satiety, suckling behavior, weight gain, and adiposity in infants [48,49,50,51]. FGF signaling and EGF receptor signaling, which are important for mammary gland development and lactation [52,53,54,55], were also among the top pathways enriched for these target genes.

In addition to delivery mode, pre-pregnancy maternal weight status, parity, and infant age at sample collection were associated with miRNA profiles in human milk EVPs. Similar to our finding that a higher maternal weight prior to pregnancy (BMI > 25 kg/m^2^) is associated with lower miRNA levels in milk, a prior study in the Faroe Islands reported inverse associations between maternal pre-pregnancy BMI and 374 different miRNAs in human milk [56]. The same study in the Faroe Islands also examined relationships between parity and infant age with milk EVP miRNA levels, although miRNAs were only assessed individually and the results were null. Interestingly, all three of these factors have also previously been associated with alterations to the human milk microbiome [46,57,58,59]. Additionally, in a study of six candidate miRNAs, miR-148a and miR-30b were both identified as having lower abundances in the milk of individuals with BMI scores above 25 kg/m^2^ prior to pregnancy relative to individuals with a BMI between 18.5 and 25 kg/m^2^ prior to pregnancy [21].

Our study has important limitations. Firstly, since this was a pilot study, we had a small sample size (N = 54), which may have limited our statistical power. Furthermore, while the NanoString nCounter method covers 98% of the high-confidence human miRNA annotated in miRbase (v22; current as of 19 January 2024), it is not comprehensive. Thus, using this method may lead to the underestimation of the total miRNA content and richness of samples if they contain rare miRNAs that are not represented on this platform. Future studies that use small RNA sequencing will, therefore, be needed to further explore these associations. Additionally, some miRNAs had very low detection rates in our samples and, therefore, could not be investigated in the current study. Future studies with larger sample sizes are, therefore, needed to examine whether detection of these very low-frequency miRNAs may also be related to maternal and pregnancy factors and subsequent child health outcomes. Our study also relied on self-reported data to derive pre-pregnancy BMI, which may have led to the misclassification of weight status prior to pregnancy due to the tendency to under-report weight [60]. This would have likely biased results toward the null. Furthermore, as our cohort reflects a predominantly rural population from New Hampshire, our findings may not be generalizable to other populations. Therefore, follow-up studies are needed to assess these associations in other populations, both within and outside the United States.

Due to the small sample size used in this pilot study, we first calculated broader measures of EVP miRNA composition as a form of dimensionality reduction, including the total number of EVP miRNA in the sample, the evenness in distribution of the relative proportions of each detected EVP miRNA (sample evenness), and the total number of unique EVP miRNA transcripts detected (sample richness). While this approach may have limited our ability to identify specific miRNA associated with each predictor of interest, it also increased our statistical power by limiting the number of tests that were run and allowed us to investigate whether any of the predictors of interest are related to broad shifts in miRNA composition, which are also highly informative. For example, shifts in sample evenness indicate that individual miRNAs are present in differing relative proportions in relation to a variable of interest, whereas associations with sample richness indicate that a variable of interest may impact overall miRNA expression or loading into EVPs.

A major strength of our study was the inclusion of paired prenatal maternal plasma and postpartum human milk samples. This allowed us to directly compare EVP miRNA profiles across these two sample types and life stages, with each reflecting potential windows for maternal–offspring transfer of miRNA. The paired design also allowed us to compare how maternal and pregnancy factors influence molecular signatures of maternal–child communication during two different and critical stages of offspring development.

Our findings suggest that plasma EVP miRNA composition may vary with the time of day of blood collection and human milk EVP miRNA may be influenced by delivery mode, maternal pre-pregnancy weight status, parity, and infant age. These results underscore the importance of considering these factors covariates in future analyses of predictors of maternal EVP miRNAs during pregnancy and postpartum periods. Given that many of these factors are known to influence child health, these findings also highlight the need for future research investigating whether EVP miRNA may mediate relationships with postpartum and child health.

## 4. Materials and Methods

### 4.1. Participant Recruitment

For this pilot study, 54 mother–infant pairs were selected from the New Hampshire Birth Cohort Study (NHBCS), which has been described previously [61]. In brief, pregnant individuals were initially recruited from prenatal clinics in New Hampshire between approximately 24 and 28 weeks of gestation who were using a private unregulated water source for their drinking water and had no plans to move during pregnancy. Inclusion criteria for the pilot samples used for this study [62] further included exclusive breastfeeding for at least 6 months, maternal submission of bilateral human milk samples at approximately 6 weeks postpartum, collection of a maternal blood sample around the time of enrollment, and availability of relevant covariate information (i.e., maternal age, pre-pregnancy BMI, smoking during pregnancy, educational attainment, infant sex, delivery mode, and gestational age at delivery). Participants also needed to have toenail metal concentrations measured, as an objective of the pilot study was to understand metal impacts on EVP miRNA [62]. All study protocols were approved by the Dartmouth College Institutional Review Board, and written informed consent was obtained from all participants upon enrollment.

### 4.2. Maternal Blood Collection and Plasma EVP miRNA Extraction

Maternal peripheral blood samples were collected at approximately 24–28 weeks gestation in K2EDTA tubes and transported at 4 °C to the Dartmouth Center for Molecular Epidemiology Biorepository for processing within 24 h. Using standard procedures, blood samples were fractionated via centrifugation, and plasma aliquots were stored at −80 °C. EVP miRNAs were extracted from 500 μL of plasma using the Qiagen Plasma/Serum exoRNeasy Kit (Qiagen, Hilden, Germany) according to the manufacturer’s protocol. EVPs extracted using this kit have been well characterized, demonstrating average diameters ranging from 100 to 600 nm [63]. Although the particles extracted from plasma are enriched for extracellular vesicles, we cannot rule out the possibility that other extracellular particles may be present and, thus, refer to them as EVPs, in accordance with MISEV guidelines [64], throughout.

Extraction efficiency was monitored using a synthetic miRNA spike-in from *Oryza sativa* (osa-miR-414; 200 pM).

### 4.3. Human Milk Collection, Processing, and EVP miRNA Extraction

As previously described [65], human milk samples were collected by participants in their homes. Participants were asked to bring 1–2 oz of expressed milk from each breast to their 6-week postpartum visit. Samples were collected in separate sterile study-collection bottles, and participants were instructed to collect the specimen after their baby’s first morning feed, within 24 h of their visit, and refrigerate the sample after collection.

Milk samples were transferred to the laboratory on ice within 24 h of collection, where they were fractioned via centrifugation at 5000× *g* rpm for 90 min at 4 °C. The supernatant fraction was aliquoted and stored at −80 °C. Milk EVP miRNAs were extracted from 500 μL of thawed supernatant samples following the manufacturer’s protocol for the Norgen Urine Exosome RNA Isolation kit (Norgen Biotek, Thorold, ON, Canada), and EVPs were characterized as previously described [62]. Although the particles extracted from milk are enriched for extracellular vesicles, as evidenced by the size range and proteomics data presented in our prior publication [62], we cannot rule out the possibility that other extracellular particles may be present and, thus, refer to them as EVPs, in accordance with MISEV guidelines [64], throughout.

The extraction efficiency of human milk EVP miRNA was monitored using a synthetic miRNA spike-in from *Oryza sativa* (osa-miR-414; 200 pM). Eluted miRNAs were further purified using Amicon Ultra Centrifugal Filters (Millipore, Billerica, MA, USA) and concentrated using a speed vacuum concentrator. The Agilent Bioanalyzer Small RNA Chip Assay (Agilent Technologies Inc., Santa Clara, CA, USA) was used for quality control and quantification of extracted miRNAs. Extracted EVP miRNA levels were then quantified by the Dartmouth Genomics Molecular Biology Shared Resource using the NanoString nCounter Expression Assay Human v3 on the nCounter Analysis System (NanoString Technologies, Inc. Seattle, WA, USA), which profiles 798 miRNAs, 100% of the high confidence human miRNA annotated to miRBase (v21) at the time of its release, and 98% of high-confidence human miRNAs annotated to miRbase (v22) as of 19 January 2024.

### 4.4. Data Processing

All data processing steps and statistical analyses were conducted in R (version 4.1.0). Raw miRNA counts obtained from the NanoString nCounter platform were normalized to sample-specific positive controls using the *NanoStringNorm* package in R (version 1.2.1.1) [66]. EVP miRNA levels were defined as above the detection threshold if they exceeded the mean + 1.5 SD of sample-specific negative controls.

### 4.5. Measures of EVP miRNA Composition

Given the pilot-sized scale of this study, we used a two-stage approach. Firstly, as a dimensionality-reduction approach, we utilized three summary measures to assess broad shifts in the compositions of miRNAs in EVPs, including two metrics commonly used for microbiome data, as EVP miRNA data are similarly zero-inflated. These measures included total miRNA counts, sample richness, and sample evenness. Total counts were calculated as the sum of the normalized counts for the 798 miRNA measured using the NanoString nCounter platform. Given that total counts were right-skewed, they were log2-transformed for downstream analyses. Sample richness was defined as the total number of unique miRNAs (of the 798 measured) that were above the detection threshold in the sample. Sample evenness [67], a measure of the distribution of levels of all detectable miRNAs in a sample, was calculated using the relative proportion (*p_i_*) of each miRNA (*i*) and the sample richness (*s*) as follows:

$$Evenness=\frac{-\sum_{i=1}^{s}p_{i}\ln\left(p_{i}\right)}{\ln\left(s\right)}$$

Sample evenness values are bound between zero and one, with a score of one reflecting all detectable EVP miRNAs being present in equal proportions. In contrast, lower sample evenness scores indicate that a small number of miRNAs are present in significantly higher abundance than other miRNA detected in the sample.

Associations between maternal and pregnancy factors and miRNA composition measures were first examined using robust linear regression with the *MASS* package in R (version 7.3-54) [68]. We then identified specific miRNAs associated with sample richness or sample evenness in each sample type. Richness and evenness were modeled as dependent variables, with miRNA above the limit of detection in 20–60% of samples modeled as binary variables (i.e., detect vs. non-detect) and miRNA above the limit of detection in more than 60% of samples modeled as continuous variables (log_2_-transformed counts). EVP miRNAs were considered significantly associated with richness or evenness if they met a pre-specified statistical significance threshold (*P_Bonferonni_* < 0.05). If a specific maternal or pregnancy factor was significantly associated with sample richness or evenness, we then conducted post hoc analyses examining that factor in relation to each of the richness- or evenness-associated miRNAs.

### 4.6. Statistical Analyses for Plasma EVP miRNA Composition

Bivariate relationships between each maternal and pregnancy factor and plasma EVP miRNA composition measure (i.e., total counts, sample richness, or sample evenness) were first evaluated to identify covariates to be included in multivariable regression models. Variables associated with any of the miRNA composition measures with a *p* < 0.1 were considered potential confounders or precision variables and, therefore, included in all subsequent models. The Spearman correlation coefficients were calculated between measures of EVP miRNA composition and continuous covariates of interest (maternal age, pre-pregnancy BMI, and gestational age at sample collection). Associations between categorical covariates (pre-pregnancy maternal weight status, parity, maternal attained education level, marital status, infant sex, and morning versus afternoon collection of blood) and measures of EVP miRNA composition were assessed using a Wilcoxon Rank-Sum (binary variables) or Kruska–Wallis test. The time of day of sample collection, maternal age, and parity (nulliparous vs. parous prior to delivery) were all found to be associated with at least one plasma EVP miRNA composition measure with a *p* < 0.10 and, therefore, included as covariates in all subsequent plasma EVP miRNA models.

Linear regression models were used to examine associations between each factor (maternal age, parity, pre-pregnancy BMI, infant sex, gestational age at sample collection, and time of day of sample collection) and each miRNA composition measure (total counts, sample richness, and sample evenness). When evaluating pre-pregnancy BMI as a categorical variable, we excluded participants with a BMI < 18.5 kg/m^2^ due to the small number of participants found in this category (n = 2).

### 4.7. Statistical Analyses for Human Milk EVP miRNA Composition

Similar to our approach for plasma, the Spearman correlation coefficients were calculated between continuous covariates of interest (maternal age, pre-pregnancy BMI, gestational weight gain, gestational age at delivery, and infant age at sample collection) and measures of milk EVP miRNA composition (i.e., total counts, sample richness, and sample evenness). Associations between categorical covariates (pre-pregnancy maternal weight status, parity, maternal attained education level, marital status, infant sex, delivery mode, and whether the milk sample was collected in the morning or afternoon) and measures of EVP miRNA composition were assessed using a Wilcoxon Rank-Sum (for binary variables) or Kruskal–Wallis test. We identified suggestive associations (*p* < 0.1) between gestational weight gain, parity (primiparous vs. multiparous after delivery), infant sex, delivery mode, and infant age at sample collection with measures of milk EVP miRNA composition; therefore, all models included these five covariates.

Linear regression models were used to assess associations between each factor (maternal age, parity, pre-pregnancy BMI, infant sex, gestational weight gain, gestational age at delivery, delivery mode, infant age at sample collection, and time of day of sample collection) and each measure of milk EVP miRNA composition (total counts, sample richness, and sample evenness). Like our analyses for plasma, analysis of pre-pregnancy BMI as a categorical variable excluded two participants with a BMI < 18.5 kg/m^2^.

### 4.8. Post Hoc Analyses Examining Individual miRNAs

If any of the maternal, pregnancy, or technical factors were found to be associated with miRNA evenness or richness in maternal plasma or milk, we conducted post hoc analyses to determine if specific miRNAs were driving these associations. These analyses were restricted to miRNAs that were significantly (*P_Bonferonni_* < 0.05) associated with the relevant composition measure. Robust linear regression was used to model the log_2_-transformed counts of EVP miRNA detectable in 60% or more of samples using the *MASS* package in R (version 7.3-54) [68]. MiRNAs detectable in 20–60% of samples were modeled as binary variables (detect vs. non-detect) using robust logistic regression with the *robustbase* package in R (version 0.93-9) [69]. Multiple testing was accounted for using *Q* values, calculated using the *qvalue* package in R (version 2.24.0), with a false discovery threshold (FDR) of *Q* < 0.05.

### 4.9. Target Gene Identification and Pathway Analysis

High-confidence target genes of EVP miRNA found to be significantly (*Q* < 0.05) associated with any of the factors examined were identified using mirDIP (version 5.2) [70]. Over-representation of these target genes in PANTHER (version 16.0) [71] pathways was investigated using the *enrichR* package in R (version 3.2) [72].

## Figures and Tables

**Table 1:** Summary statistics for participant and biospecimen characteristics (n = 54).

Demographic	Mean (SD) or n (%)
**Maternal Age** (yrs)	32.6 (4.0)
**Maternal Education**	
High School Graduate or Less	4 (7.4)
Some College	3 (5.6)
College Graduate or More	47 (87.0)
**Maternal Relationship Status**	
Married	45 (83.3)
Separated or Never Married	9 (16.7)
**Maternal Pre-Pregnancy BMI** (kg/m^2^)	23.9 (4.9)
**Maternal Pre-Pregnancy Weight Status**	
BMI < 18.5 kg/m^2^	3 (5.6)
BMI 18.5–25 kg/m^2^	39 (72.2)
BMI > 25 kg/m^2^	12 (22.2)
**Gestational Weight Gain** (lbs)	34.8 (18.9)
**Infant Sex**	
Female	30 (55.6)
Male	24 (44.4)
**Gestational Age at Delivery** (wks)	39.2 (1.5)
**Delivery**	
Preterm (<37 weeks)	6 (11.1)
Term (≥37 weeks)	48 (88.9)
**Delivery Mode**	
Vaginal	40 (74.1)
Cesarean Section	14 (25.9)
**Gestational Age at Plasma Collection** (wks)	28.8 (2.8)
**Time of Day of Plasma Collection**	
Morning (7:00 AM–12:00 PM)	27 (50.0)
Afternoon (12:00 PM–6:00 PM)	27 (50.0)
**Infant Age at Milk Collection** (wks)	6.1 (0.8)
**Time of Day of Milk Collection** ^a^	
Morning (12:00 AM–12:00 PM)	40 (76.9)
Afternoon (12:00 PM–12:00 AM)	12 (23.1)

^a^ Two subjects failed to report the time of day of milk collection.

**Table 2:** Associations between maternal, pregnancy, and sample collection factors and plasma EVP miRNA composition.

	Total Counts ^a^		Richness		Evenness	
	Effect Size (95% CI)	*p* Value	Effect Size (95% CI)	*p* Value	Effect Size (95% CI)	*p* Value
**Maternal Age** (yrs)	0.003 (−0.022, 0.028)	0.824	−3.463 (−8.636, 1.709)	0.195	−0.002 (−0.009, 0.005)	0.517
**Parity** ^b^						
Nulliparous	Ref.	Ref.	Ref.	Ref.	Ref.	Ref.
Parous	0.157 (−0.047, 0.361)	0.137	18.096 (−23.588, 59.780)	0.399	0.057 (0, 0.114)	0.056
**Pre-Pregnancy BMI** (kg/m^2^)	−0.001 (−0.022, 0.019)	0.887	−0.294 (−4.432, 3.844)	0.890	0.001 (−0.005, 0.007)	0.701
**Pre-Pregnancy Weight Status** ^c^						
BMI 18.5–25 kg/m^2^	Ref.	Ref.	Ref.	Ref.	Ref.	Ref.
BMI > 25 kg/m^2^	0.006 (−0.218, 0.229)	0.961	15.197 (−34.074, 64.468)	0.548	0.036 (−0.031, 0.103)	0.301
**Infant Sex**						
Female	Ref.	Ref.	Ref.	Ref.	Ref.	Ref.
Male	−0.096 (−0.294, 0.103)	0.349	−6.903 (−47.798, 33.992)	0.742	0.002 (−0.054, 0.058)	0.945
**GA at Blood Collection** (days)	0.001 (−0.004, 0.006)	0.720	0.405 (−0.614, 1.423)	0.440	0 (−0.001, 0.002)	0.866
**Time of Day of Blood Collection**						
7 AM–12 PM	Ref.	Ref.	Ref.	Ref.	Ref.	Ref.
12 PM–6 PM	0.234 (0.037, 0.430)	0.024	8.693 (−31.48, 48.867)	0.673	−0.093 (−0.148, −0.038)	0.002

Each miRNA composition measure was modeled separately as the dependent variable using linear regression. All models were adjusted for maternal age (continuous), parity (binary), and the time of day of sample collection (binary). ^a^ Effect sizes and 95% confidence intervals reflect the changes in log_2_-transformed total EVP miRNA counts. ^b^ Assessed at the time of plasma collection. ^c^ n = 52.

**Table 3:** Associations between maternal, pregnancy, and sample collection factors and milk EVP miRNA composition.

	Total Counts ^a^		Richness		Evenness	
	Effect Size (95% CI)	*p* Value	Effect Size (95% CI)	*p* Value	Effect Size (95% CI)	*p* Value
**Maternal Age** (yrs)	−0.034 (−0.099, 0.031)	0.307	−1.855 (−5.237, 1.527)	0.288	0 (−0.003, 0.004)	0.806
**Parity** ^b^						
Primiparous	Ref.	Ref.	Ref.	Ref.	Ref.	Ref.
Multiparous	−0.526 (−1.032, −0.019)	0.047	−17.262 (−43.69, 9.166)	0.207	0.014 (−0.014, 0.041)	0.331
**Pre-Pregnancy BMI** (kg/m^2^)	−0.032 (−0.086, 0.023)	0.257	0.711 (−2.155, 3.576)	0.629	0.001 (−0.002, 0.004)	0.704
**Pre-Pregnancy Weight Status** ^c^						
BMI 18.5–25 kg/m^2^	Ref.	Ref.	Ref.	Ref.	Ref.	Ref.
BMI > 25 kg/m^2^	−0.645 (−1.232, −0.058)	0.037	−13.548 (−45.98, 18.884)	0.417	0.022 (−0.012, 0.055)	0.209
**Infant Sex**						
Female	Ref.	Ref.	Ref.	Ref.	Ref.	Ref.
Male	0.389 (−0.124, 0.901)	0.144	22.036 (−4.706, 48.777)	0.113	−0.018 (−0.045, 0.01)	0.219
**Gestational Weight Gain** (lbs)	0.009 (−0.005, 0.023)	0.196	0.48 (−0.233, 1.193)	0.193	−0.001 (−0.001, 0)	0.120
**Gestational Age at Birth** (wks)	0.034 (−0.151, 0.218)	0.723	−0.169 (−9.811, 9.474)	0.973	−0.001 (−0.011, 0.009)	0.870
**Delivery Mode**						
Vaginal	Ref.	Ref.	Ref.	Ref.	Ref.	Ref.
Cesarean Section	−0.703 (−1.28, −0.125)	0.021	−14.626 (−44.762, 15.51)	0.346	0.040 (0.009, 0.071)	0.015
**Infant Age at Milk Collection** (days)	−0.049 (−0.095, −0.004)	0.040	−0.655 (−3.039, 1.73)	0.593	0 (−0.003, 0.002)	0.774
**Milk Collection Time** ^b^						
12:00 AM–12:00 PM	Ref.	Ref.	Ref.	Ref.	Ref.	Ref.
12:00 PM–12:00 AM	0.21 (−0.412, 0.833)	0.511	13.052 (−19.668, 45.771)	0.438	−0.012 (−0.047, 0.022)	0.481

Each miRNA composition measure was modeled separately as the dependent variable using linear regression. All models were adjusted for infant age at sample collection (continuous), gestational weight gain (continuous), parity (binary), infant sex (binary), and delivery mode (binary). ^a^ Effect sizes and 95% confidence intervals reflect the changes in log_2_-transformed total EVP miRNA counts. ^b^ Assessed at the time of milk collection. ^c^ n = 52.

## Data Availability

EVP miRNA data are available under the Gene Expression Omnibus (GEO) accession codes GSE223272 (milk samples) and GSE223273 (plasma samples).

## Supplementary Materials

The following supporting information can be downloaded via this link: https://www.mdpi.com/article/10.3390/ijms25031538/s1.
