# Supplementary material for: Maternal–Infant Factors in Relation to Extracellular Vesicle and Particle miRNA in Prenatal Plasma and in Postpartum Human Milk
Source: Int J Mol Sci. 2024 Jan 26;25(3):1538. doi: 10.3390/ijms25031538 (PMC10855220; doi:10.3390/ijms25031538)
Supplement: Supplementary file 1 [file ijms-25-01538-s001.zip › DescriptiveEVPmiRNA_SupplementalFigures.docx]

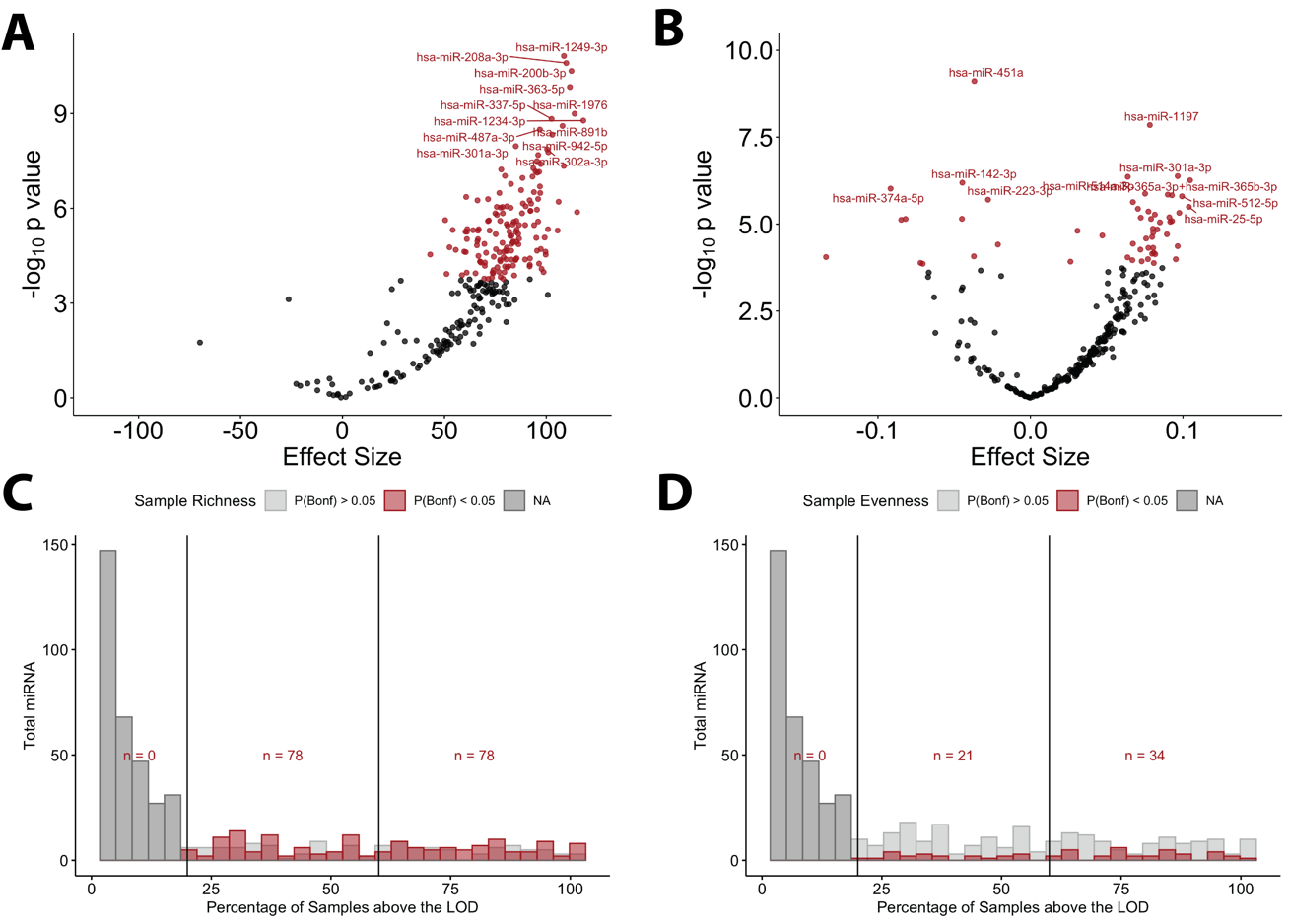


**Figure S1**: MiRNA in plasma EVPs associated with sample (**A**) richness and (**B**) evenness. Associations were modeled using robust linear regression treating miRNA counts as the independent variable. MiRNA with levels above the limit of detection for 20-60% of samples were treated as binary variables (detect vs non-detect) and miRNA above the limit of detection for more than 60% of samples were treated as continuous variables (log_2_-transformed counts). Red points reflect a significance of *P_Bonferonni_* < 0.05. Distribution of miRNA associated with (**C**) richness and (**D**) evenness by the proportion of samples in which the miRNA was above the limit of detection. N/A indicates miRNA above the limit of detection in less than 20% of samples which were not included in the analysis.


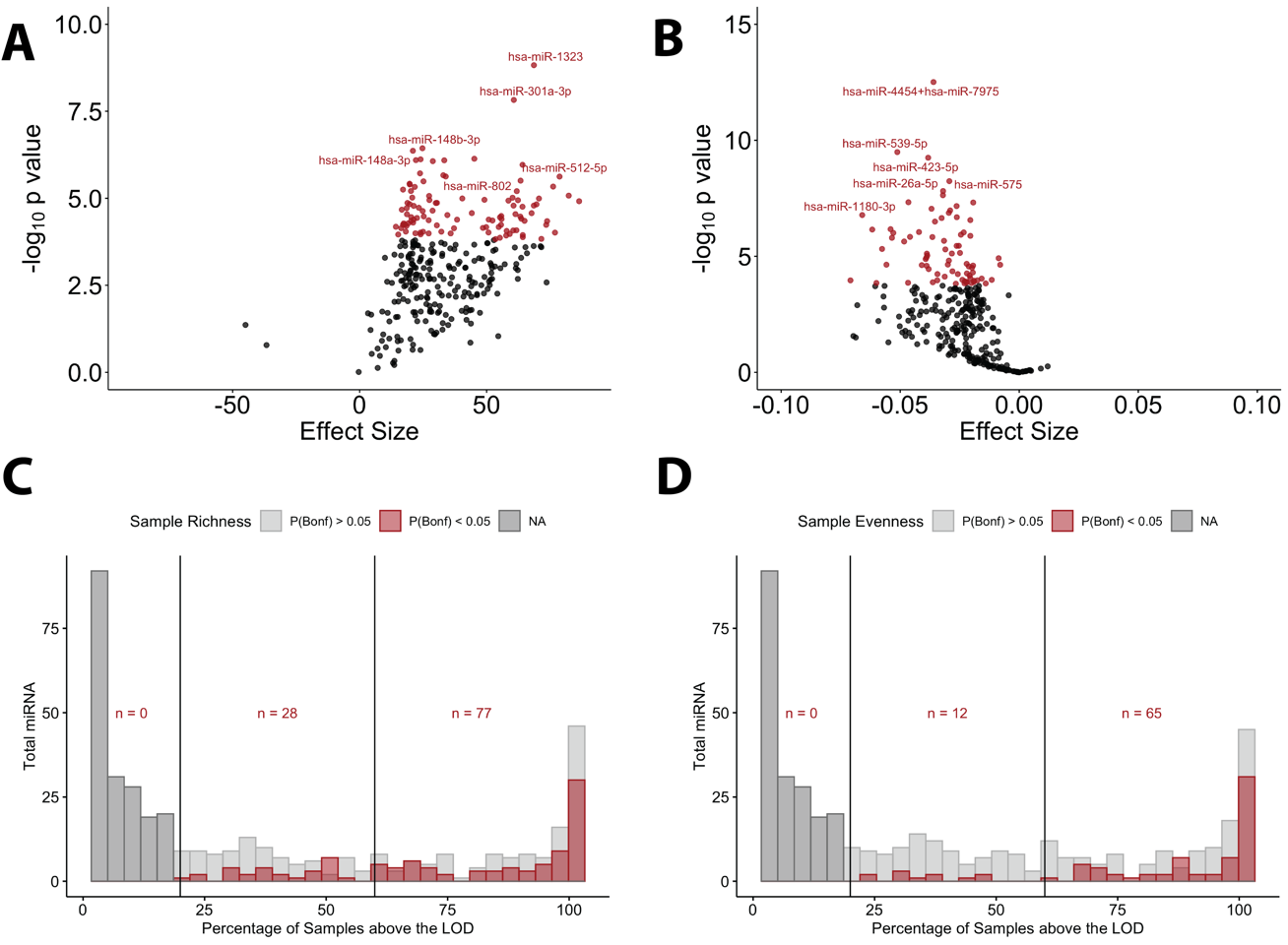


**Figure S2**: MiRNA in milk EVPs associated with sample (**A**) richness and (**B**) evenness. Associations were modeled using robust linear regression treating EVP miRNA counts as the independent variable. EVP miRNA with levels above the limit of detection for 20-60% of samples were treated as binary variables (detectable vs. non-detectable) and miRNA above the limit of detection in more than 60% of samples were treated as continuous lvariables (log2-transformed counts). Red points reflect a significance of *P_Bonferonni_* < 0.05. Distribution of miRNA associated with (**C**) richness and (**D**) evenness by the proportion of milk samples in which the miRNA was above the limit of detection. N/A indicates miRNA above the limit of detection in less than 20% of samples which were not included in the analysis.


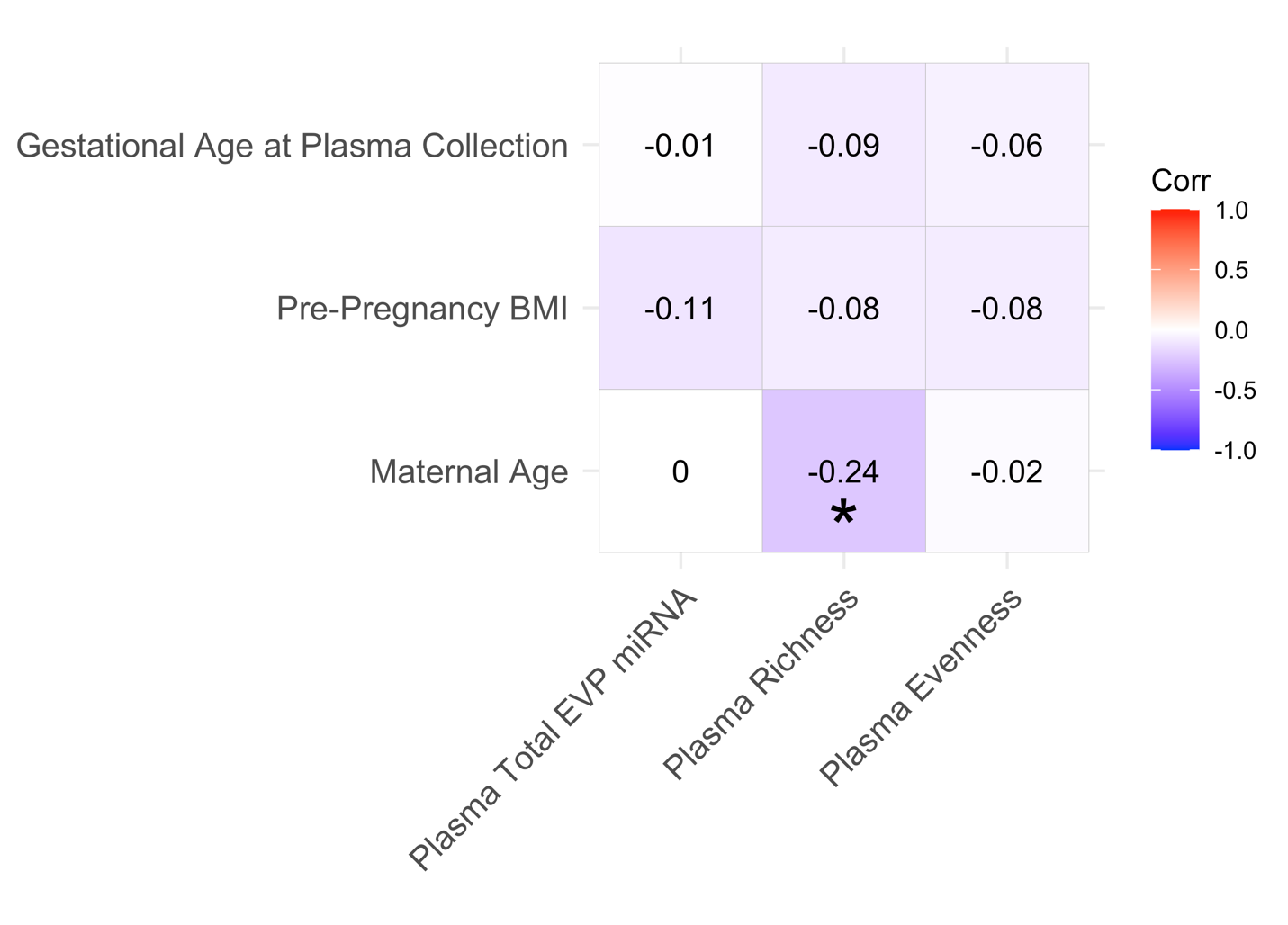


**Figure S3** Spearman correlation coefficients between continuous maternal and pregnancy factors and measures of plasma EVP miRNA composition. Correlations with * indicate suggestive statistical significance (0.05 ≤ *P* < 0.1).


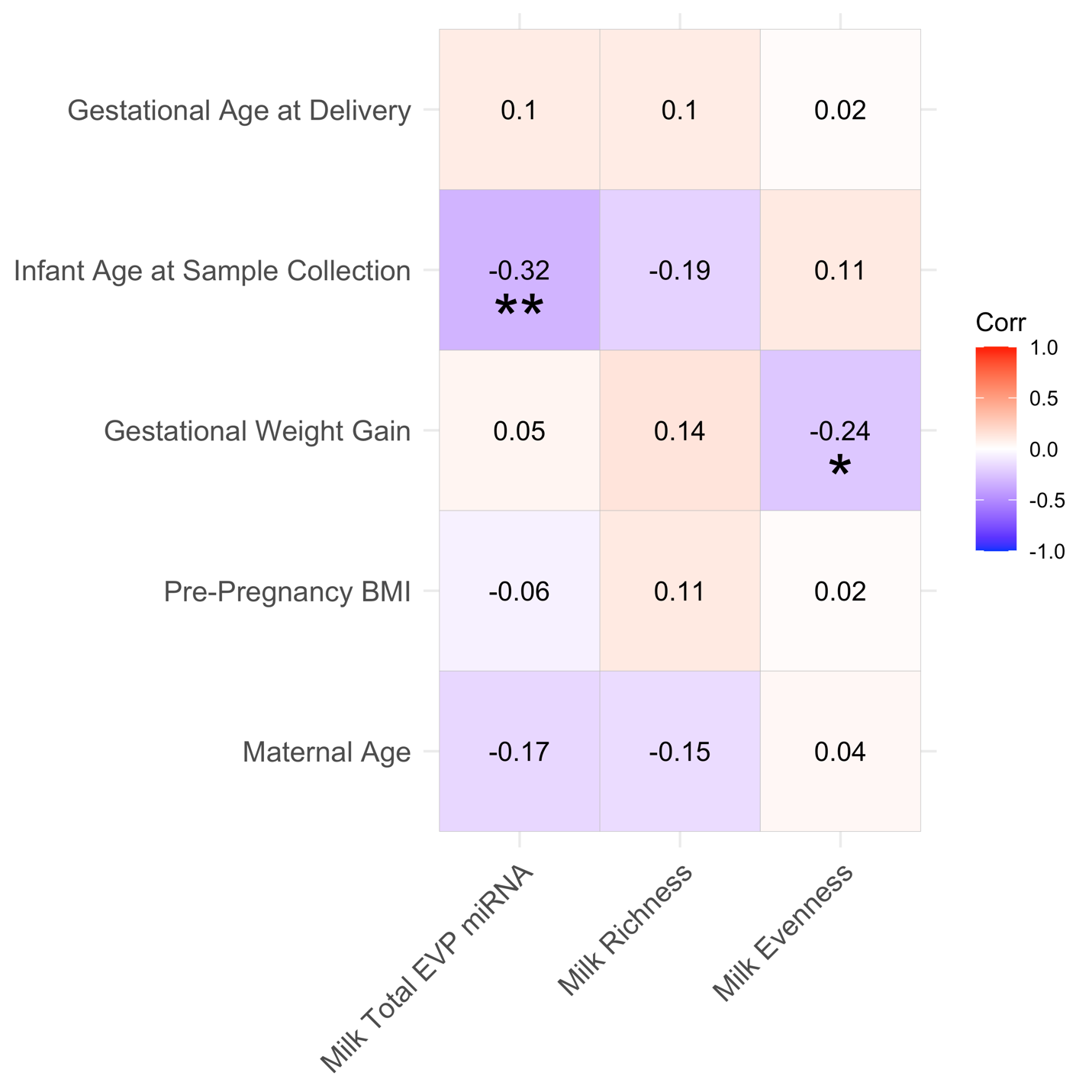


**Figure S4** Spearman correlation coefficients between continuous maternal and pregnancy factors and measures of milk EVP miRNA composition. Correlations with ** indicate statistical significance (*P* < 0.05) and those marked with * indicate suggestive statistical significance (0.05 ≤ *P* < 0.1).
